# What is my walking neighbourhood? A pilot study of English adults' definitions of their local walking neighbourhoods

**DOI:** 10.1186/1479-5868-7-34

**Published:** 2010-05-06

**Authors:** Graham Smith, Christopher Gidlow, Rachel Davey, Charles Foster

**Affiliations:** 1 Institute for Environment, Sustainability and Regeneration, Staffordshire University, Mellor Building, College Road, Stoke on Trent, ST4 2DE, UK; 2 Centre for Sport, Health and Exercise Research, Staffordshire University, Stoke on Trent, UK; 3 Centre for Research and Action in Public Health, Faculty of Health, University of Canberra, ACT 2601, Australia; 4 British Heart Foundation Health Promotion Research Group, Department of Public Health, University of Oxford, Oxford, UK

## Abstract

**Background:**

Existing measures of perceptions of the environment associated with walking commonly rely on providing a definition of 'neighbourhood', e.g. 1 mile area around the home. We have little understanding of how these examples relate with adults' own geographical definitions of their neighbourhood area. Our pilot study examined the congruence between definitions used in environmental questionnaires and adults' own definitions of neighbourhood.

**Methods:**

We conducted 58 face-to-face interviews with participants randomly selected from 10 areas of Stoke-on-Trent, England. Participants were shown printed maps showing their local area with road names and places of interest (e.g. shops, services, green space) and were asked: (i) to recall usual walking destinations (from their home); (ii) to draw their 'neighbourhood walking area' on the map. Annotated maps were scanned back into GIS for analysis.

**Results:**

When asked to draw their 'neighbourhood' boundary, the resulting area drawn by participants on average represented only 16 ± 20% of the commonly used total straight-line buffer of 1 mile (or 1.6 km) with a range of 0.3% to 111%. Even when repeated using a network buffer (rather than straight-line) the same comparison resulted in a mean of 36% (± 47%) and a range of 0.6 to 245%.

**Conclusions:**

We found that adults' interpretation of their neighbourhood area does not appear to relate accurately to the definitions typically used in research into environmental perceptions and walking. This mis-match warrants further investigation as definitions used in existing measures may be consistently misclassifying perceived local walking neighbourhoods.

## Introduction

Policy makers are increasingly recognising that creating environments to encourage healthy behaviours and discourage unhealthy behaviours may help to reduce health inequalities [1]. This recognition has seen the introduction of legislative and environmental interventions to shape choices across a number of delivery sectors including transport, education and urban planning [2]. The negative impact of the built environment has been seen as a contributing factor to non-communicable disease, including cardio-vascular disease, cancer and obesity [1,3,4]. This impact has focused research to examine the relationship between the environment and different risk factors (e.g. physical inactivity) using the socio-ecological models of behaviour [5].

Growth in environmental studies examining the socio-ecological correlates of physical activity reflects policy makers' desire to create environments that enable people to build physical activity into their daily lives [2,6]. There is evidence that much of our daily physical activity is accumulated through the activities of daily living, such as walking for transport, work and domestic activity, rather than through active leisure pursuits [7,8]. In the UK walking is consistently found to be the most popular type of physical activity across all strata of activity levels [8]. A recent systematic review identified walking as an ideal health behaviour given its broad accessibility, convenience, the lack of associated cost or need for equipment, and sustainability into old age [9]. To create 'walkable' environments, we need to understand how people's local 'neighbourhood' environment impacts on their walking behaviour [10].

Studies in this field have either examined the associations between overall or specific categories of physical activity and perceptions of the environment, or objectively measured environmental variables [11,12]. The challenge of measuring the 'neighbourhood environment' has been helped by the use of Geographical Information Systems (GIS) [13]. The emergence of GIS in physical activity research enables objective measurement of the environment, linking geographical and epidemiological information through spatially locating socio-demographic, behavioural and environmental data.

Research into perceived environment and physical activity is also rapidly expanding and different environmental measures have been developed, mostly in the US and Australia [14]. Spittaels and colleagues' systematic review identified a number of inconsistencies between existing measures of perceptions of the environment [14]. Definitions in existing measures ranged from such vague spatial formulations as 'neighbourhood' and 'local area' to more behavioural definitions 'within a 5 to 10 minute walk'. One key issue was to have clearly defined neighbourhood and area properties that are cogent with residents' definitions. The review found numerous tools to capture important perceived environmental characteristics to explore possible links between walking behaviour and 'neighbourhood' [15,16].

'Neighbourhood', however, can have different connotations depending on an individual's interpretation [17]. It can be defined at different scales and by various characteristics depending on context and purpose [18]. In relation to walking, an operational definition is yet to be agreed [19].

One means of exploring peoples' perceived neighbourhood is through mental mapping. This involves individuals drawing the boundaries of their neighbourhood and has been used to explore variation in, and potential factors that influence, how people define their own neighbourhood area [17]. In physical activity research, however, the congruence of such individually defined neighbourhood areas and those typically used in ecological walking and physical activity research remains unknown. The potential value of using these methods in combination with surveys that explore specific destinations people walk to and objective environmental measures has been recognised elsewhere [20].

This pilot study combined mental mapping and GIS to examine the congruence between definitions used in environmental questionnaires and adults' own definitions of their neighbourhood, whilst testing the feasibility of the approach described.

## Methods

Face-to-face interviews lasting 20-30 minutes were conducted with adults (aged 20-65 years) from an existing cohort randomly selected from 10 urban areas in Stoke-on-Trent, England [7]. For each of the 10 Lower Level Super Output Areas in the city (LSOA; mean population 1500) [21] GIS maps were created using 1:10,000 scale Ordnance Survey Street View mapping. Maps displayed an area of 1 mile around the boundary of each LSOA (approximately centred on the participants' residence) and included road names and places of local interest such as green space, shops, services, schools and physical activity facilities.

Each participant was shown the printed GIS map for their local area. The interviewer helped to orientate them by pointing out the location of their residence, main roads, and local landmarks. Using maps for reference or simply from recall, participants were asked to:

(i) Recall all recent (last seven days) and usual walking destinations from their home. In the event that participants were unable to identify any (more) walking destinations, interviewer prompts were used to ask them to recall any places they had walked from their home without using the map, or used the map to identify possible destinations (e.g., local shopping areas, pubs/bars, family/friends).

(ii) Draw their 'neighbourhood area' on the map. Participants were advised that it could be any size or shape, and that there was no right or wrong answer.

Annotated maps from all participants were scanned back into a GIS for analysis. All recalled destination points and 'neighbourhood area' boundaries were digitised and the annotation from the maps recorded as feature attributes. GIS analysis was used to create a number of Euclidean and network distance buffers around the address location of each participant. A Euclidean buffer is a straight line circular radius around an address, whereas the network buffers were calculated by measuring a defined distance along the pedestrian street network (i.e., roads and pathways used by pedestrians) in all possible directions away from a participant's address. The end points of these routes were joined together to form an enclosed area representing the total area within a defined walking distance of the address.

**Figure 1:**
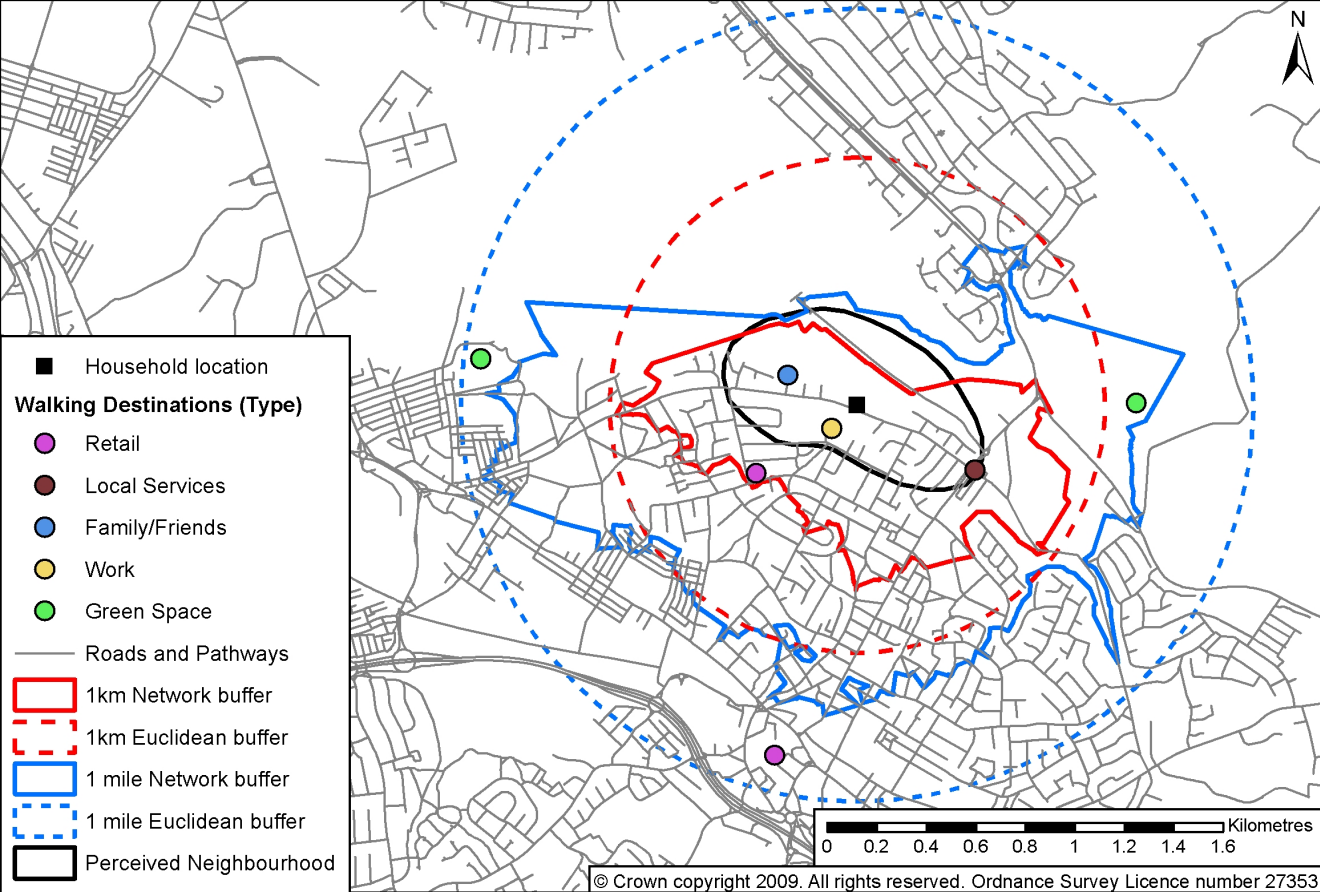
**Illustrative figure of GIS-defined and perceived neighbourhood boundaries and reported walking destinations**.

For each participant we produced five different neighbourhood areas (Figure 1): 1 mile (or 1.6 km) around the individual's home, network and Euclidean buffers; 1 km around the individual's home, network and Euclidean buffers; participant's perceived neighbourhood area. The areas of the perceived neighbourhoods were calculated as a proportion of the different network and Euclidean buffers to explore discrepancies in area size [i.e., area within perceived neighbourhood boundary (m^2^)/area within network or Euclidean neighbourhood boundary (m^2^) × 100].

Reported walking destinations were placed into eight categories used in the recently developed European ALPHA questionnaire [14]: retail (e.g., shops, supermarkets, grocers); local services (e.g., banks, libraries); eating and drinking (e.g., pubs, cafes, restaurants); family and friends; work/school; bus stops; green space (e.g., parks and common areas); and physical activities facilities (e.g., leisure centres, private gyms, swimming pools). Each geocoded destination was defined as lying *within *or *outside *each of the five different neighbourhood areas. These were examined for the sample as a proportion of total walking destinations.

The study was approved by the Staffordshire University ethics committee.

## Results

Out of 176 potential participants, 70 agreed to take part. Complete data from 58 individuals were included in the present analyses, 31 men and 27 women of mean age 42.5 ± 10.8 years; most had lived in the area for many years (mean 17.7 ± 14.7 years). Seven out of the 10 areas in which participants resided fell within the most deprived 40% of national deprivation rankings, with none in the top 40% [22]. This was consistent with the low socio-economic position evident from individual-level socio-economic indicators (25.9% had no formal qualifications; 41.4% in routine/manual occupations), although most participants had access to a car (81.0%).

**Figure 2:**
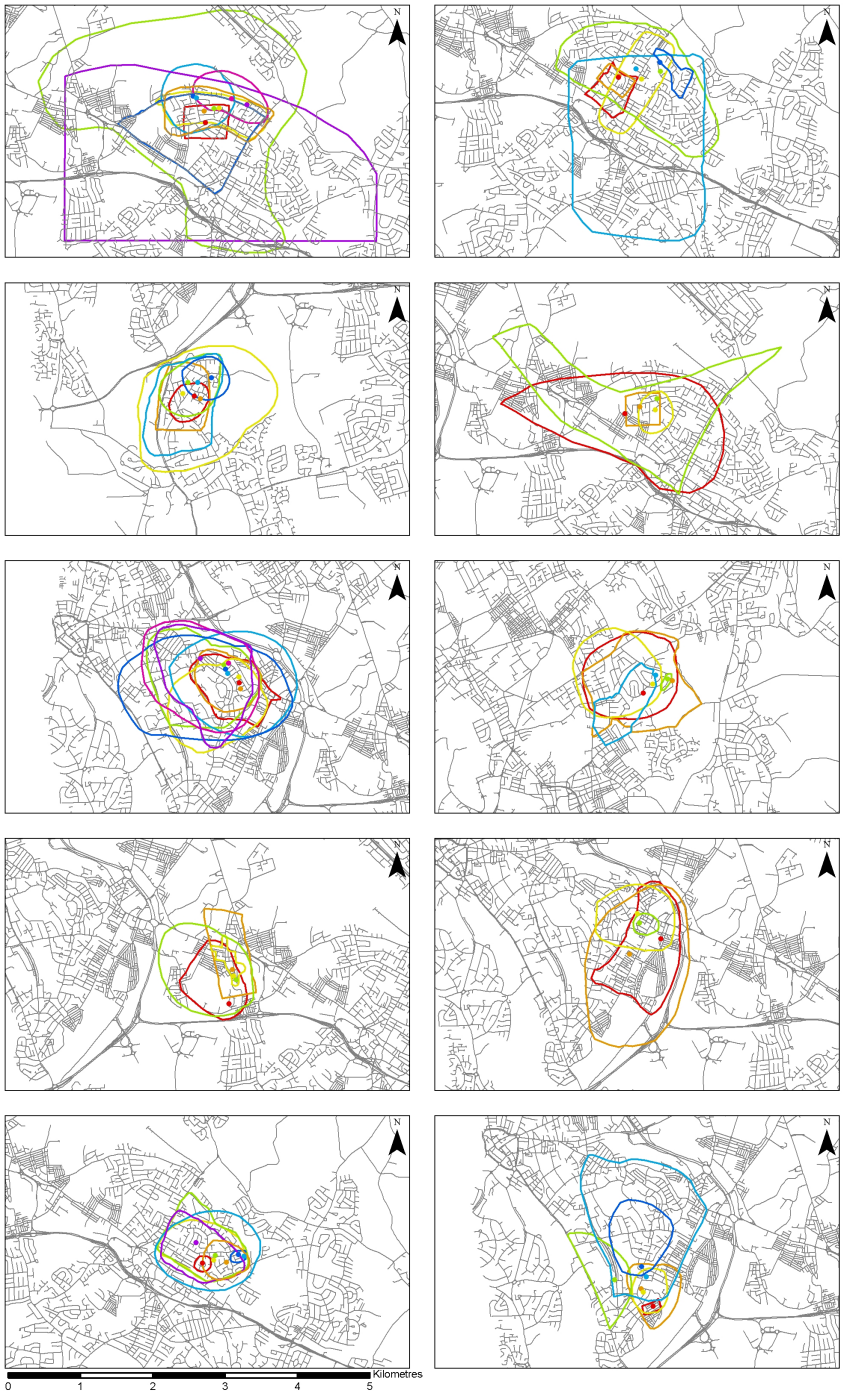
**Different conceptual neighbourhoods drawn by participants across 10 urban areas (n = 58)**. These maps display the conceptual neighbourhoods drawn by participants and the location of the participants' homes. Within each map the home location point and neighbourhood area of a participant are drawn in the same colour.

When participants were asked to draw their 'neighbourhood' boundary, the resulting areas were smaller than those calculated using GIS based on standard neighbourhood definitions (1 mile or 1 km around the home). *Perceived *neighbourhood areas, on average, represented only 16 ± 20% (range of 0.3% to 111%) of the commonly used 1 mile (1.6 km) *Euclidean *buffer area. Compared with a 1 mile *network *buffer area, the perceived neighbourhood area accounted for just 36 ± 46% (range 0.6 to 245%). This indicated a large discrepancy and considerable variation in relative size of perceived versus standard 1 mile neighbourhood definition. Analyses repeated using 1 km buffer areas revealed that, on average, the perceived neighbourhood still represented less than the Euclidean (41 ± 51%; range 0.6 to 284%) and network buffer areas (96 ± 123%; range 2 to 691%). The mean of 96% is somewhat misleading as the 1 km network buffer was at least twice the size of the neighbourhood area in 64% of cases. Figure 2 shows the range of differently drawn neighbourhood areas for participants in each of the 10 areas.

Table 1 summarises counts and proportions of the sample reporting different walking destinations. Most residents reported walking to retail destinations, approximately half walked to local green space and over one-third to family/friends and eating/drinking establishments. This pattern was similar when looking at the contribution of each destination type as a proportion of the total number reported. Only three participants reported walking to physical activity facilities, whereas informal recreational activity through visiting green space was more frequently reported.

**Table 1:** Number and percentage of participants who walked to different destination types (n = 58)

Destination Type	Resident		Destination	
	
	n	%	n	%
Retail	52	89.7	119	40.8
Local Services	17	29.3	23	7.9
Eating and Drinking	22	37.9	43	14.7
Family/Friends	21	36.2	33	11.3
Work/School	18	31.0	24	8.2
Bus Stop	3	5.2	4	1.4
Green Space	27	46.6	43	14.7
PA Facility	3	5.2	3	1.0
Total	58		292	

Table 2 highlights the discrepancies between the number (and percentage) of walking destinations that fell within the variously defined neighbourhood areas. The commonly used 1 mile Euclidean buffer area captured over 95% of destinations, with the network buffer capturing marginally less. A more marked difference was apparent between Euclidean and network buffer areas for 1 km. Given the size difference, the neighbourhood areas calculated using questionnaire definitions (i.e., 1 mile or 1 km around the home) captured more destinations than the participants' perceived neighbourhood areas; 42% of all destinations reported were outside perceived neighbourhood areas.

**Table 2:** Number and percentage of participants who walked to destinations types within perceived and objectively defined neighbourhood (1 km and 1 mile) (n = 58)

Neighbourhood definition	Total destinations for sample	Mean number reported per person	% of total destinations	Mean % of destinations per person	Resident count	%
1 km buffer:						
*Euclidean*	254	4.54	87.0	86.05	56	96.55
*Network*	209	3.80	71.58	72.70	55	94.83
1 mile buffer:						
*Euclidean*	281	5.02	96.23	93.79	56	96.55
*Network*	264	4.71	90.41	88.76	56	96.55
'Perceived' neighbourhood	169	3.38	57.88	61.14	50	86.21

## Discussion

We used mental mapping and GIS to explore congruence between peoples' perception of their neighbourhood area and definitions typically used in studies examining the association between environmental variables and physical activity.

Our results highlight important discrepancies depending on the neighbourhood definition used, with participants generally conceptualising a smaller neighbourhood area than those typically used in questionnaires. Perceived neighbourhood areas tended to be considerably smaller than those often used in physical activity research, from 1 km (or 0.5 mile) Euclidean buffer areas [23,24] to those of 1 mile or more [15,25-27]. Colabianchi reported that a 0.75 mile buffer was appropriate to define the walking neighbourhood within "easy walking distance" for older female adolescents [28]. Our sample were adults, the majority of whom had access to a car for personal use and whose low levels of transport-related activity had been previously demonstrated [7]. It is, therefore, reasonable that the average walkable neighbourhood area in our sample would be smaller than 0.75 miles (as the data infer) and closer to estimated '5-minute walking' distances used elsewhere (e.g., 0.25 miles [29], 400 m [30]). In this sense, it would appear that operational definitions of neighbourhood need to be smaller than those typically used.

On the other hand, analysis of the destinations showed that as a result of this size discrepancy, 42% of all walking destinations fell outside of areas that participants perceived as 'their neighbourhood'. It could, therefore, be argued that use of the larger 1 mile Euclidean buffer, which captured 96% of destinations, is acceptable despite its lack of congruence with the perceived area.

Although important for developing tools to measure environmental perceptions, the absolute size of the area to apply might not be the important issue. There was great variety in the size of individuals' perceived neighbourhoods, ranging from single streets, to areas including the local town centre and surround. This variation is likely to pose a greater problem. Regardless of size, imposing a simple uniform definition in studies of environmental perceptions should promote comparability and help to standardise the areal boundaries for what is a very subjective process (i.e. judging the presence, proximity or quality of characteristics within a given area). Yet there are lessons from the sociology literature that have yet to translate into environmental physical activity research [20]. Mental mapping exercises by Chaskin [17] have shown that neighbourhood could be defined as a social unit, a spatial unit, or a network of relationships, associations and patterns of use, and that this has implications for size. For example, those defining their neighbourhood in terms of social relationships are more likely to describe smaller units, than those thinking of institutions and other frequently travelled destinations. Moreover, while individuals might stress one dimension over another, the area is rarely the result of a single dimension. Moudon and colleagues [19] who explored some of these concepts within the context of walking behaviour, stated that, 'Neighbourhood evokes socio-physical homogeneity, a shared sense of place, connection, and access. It has multiple cognitive, economic, geographic, behavioural, cultural, and temporal dimensions' (p.S102). This multi-factorial nature of defining your own neighbourhood could explain the marked variation in size and shape observed in the present study and reported elsewhere [20]. It highlights the complexity of the neighbourhood concept and the challenge of measurement.

We were not surprised that the most popular local walking destinations were shopping/retail destinations. However, the importance of family and friends as a destination reported in 36% of participants suggests that it should be a feature of neighbourhood environment-walking/physical activity surveys. To date, it has been largely ignored, with few exceptions [31,32]. Half of participants reported walking to green space, compared with just 5% who walked to physical activity facilities. The importance of informal recreation and access to quality green space has been reported in previous UK studies [7,33-35] and appeared to be confirmed by the data presented.

Our findings have identified a number of issues that warrant further consideration by researchers. We need to better understand what people are thinking when we ask them questions about their neighbourhood environment. Despite offering standard definitions, the multitude of potentially influential social and cultural factors, both individual and areal, in addition to the context of the question (e.g. whether asking about the presence of trees on streets or pedestrianised areas), clearly results in wide inter-individual variation in the size of neighbourhood area. When developing surveillance tools, it will not be possible to take all of these factors into account, but further work to reach manageable data collection processes that improve on current practice in physical activity research is certainly warranted.

At least in relation to transportation walking, we have highlighted an opportunity to further explore walking destinations and the perceptions of the environment *en route *as a potential alternative or adjunct to 'neighbourhood' in physical activity and walking studies. The approach piloted here used maps with a level of detail that appeared fit for purpose and manageable from both researcher and participant perspectives. But this was to record the location of destinations only. To gather information on each of the routes (and there could be several for each destination, and many destinations) would represent a hugely time consuming and detailed process, greatly increasing participant burden. Therefore, the logistics of comprehensively capturing this information would be prohibitive for monitoring and surveillance, but there is scope for further work to turn this concept into a manageable and simplified, but valid data collection process.

The findings also make a case for similar work on a larger scale, including more qualitative evaluation, and for referring to other disciplines where such issues have been researched in more depth. By using lessons learned from sociological investigations, and using relevant approaches such as mental mapping, GIS, Global Positioning Systems (GPS) and cognitive interviewing, our understanding of this important area can be improved. Our study sample was too small to be able to identify any patterns of conceptual definition or neighbourhood destinations, which we would recommend for future investigation. We did observe much confusion and different abilities amongst our sample to identify their home location and neighbourhood on their maps, a further limitation of this approach. However, consistent with the views of others [20], we feel the feasibility and novel data produced, warrant pursuing and refining this approach in an effort to reduce the potential misclassification of local walking neighbourhoods.

## Conclusions

We found that adults' interpretation of their neighbourhood area does not appear to relate accurately to the definitions typically used in research into environmental perceptions and walking. By achieving greater precision in our measures and including qualitative research methodology, we can better identify the aspects of the environment most important for different physical activities for different population groups.

## Competing interests

The authors declare that they have no competing interests.

## Authors' contributions

GS, CG and CF conceived the study, collected data, conducted the analysis and drafted the manuscript. RD participated in its design and coordination and helped to draft the manuscript. All authors read and approved the final manuscript.
